# Can scrotal circumference-based selection discard bulls with good productive and reproductive potential?

**DOI:** 10.1371/journal.pone.0193103

**Published:** 2018-03-01

**Authors:** Jurandy Mauro Penitente-Filho, Faider Alberto Castaño Villadiego, Fabyano Fonseca e Silva, Breno Soares Camilo, Victor Gomez León, Thiago Peixoto, Edgar Díaz, Denise Okano, Paula Maitan, Daniel Lima, Simone Facioni Guimarães, Jeanne Broch Siqueira, Rogério Pinho, José Domingos Guimarães

**Affiliations:** 1 Department of Veterinary, Universidade Federal de Viçosa, Viçosa, Minas Gerais, Brazil; 2 Department of Animal Science, Universidade Federal de Viçosa, Viçosa, Minas Gerais, Brazil; 3 Institute of Agricultural Sciences, Universidade Federal dos Vales do Jequitinhonha e Mucuri, Unaí, Minas Gerais, Brazil; Universite Clermont Auvergne, FRANCE

## Abstract

Nonlinear mixed models were used to describe longitudinal scrotal circumference (SC) measurements of Nellore bulls. Models comparisons were based on Akaike’s information criterion, Bayesian information criterion, error sum of squares, adjusted R^2^ and percentage of convergence. Sequentially, the best model was used to compare the SC growth curve in bulls divergently classified according to SC at 18–21 months of age. For this, bulls were classified into five groups: SC < 28cm; 28cm ≤ SC < 30cm, 30cm ≤ SC < 32cm, 32cm ≤ SC < 34cm and SC ≥ 34cm. Michaelis-Menten model showed the best fit according to the mentioned criteria. In this model, *β*_*1*_ is the asymptotic SC value and *β*_*2*_ represents the time to half-final growth and may be related to sexual precocity. Parameters of the individual estimated growth curves were used to create a new dataset to evaluate the effect of the classification, farms, and year of birth on *β*_*1*_ and *β*_*2*_ parameters. Bulls of the largest SC group presented a larger predicted SC along all analyzed periods; nevertheless, smaller SC group showed predicted SC similar to intermediate SC groups (28cm ≤ SC < 32cm), around 1200 days of age. In this context, bulls classified as improper for reproduction at 18–21 months old can reach a similar condition to those considered as good condition. In terms of classification at 18–21 months, asymptotic SC was similar among groups, farms and years; however, *β*_*2*_ differed among groups indicating that differences in growth curves are related to sexual precocity. In summary, it seems that selection based on SC at too early ages may lead to discard bulls with suitable reproductive potential.

## Introduction

Scrotal circumference (SC) is commonly used in breeding programs due to its easy of measurement, high repeatability, and moderate to high heritability which varies from 0.36 to 0.69 [1–4]. Besides, SC is related to testis development in bulls [5]; and it is associated with age at puberty of males and females [6, 7]. The SC shows positive genetic correlations with reproductive characteristics in females such as heifer pregnancy and stayability [8]; thus, selection based on SC has a positive influence on reproductive performance of females [9, 10].

Since SC is considered a useful tool for predicting age at puberty [11], the selection of bulls based on this trait at earlier ages is performed to improve the reproductive performance of the herd [4]. In several breeding programs of the Nellore bulls, the SC has been measured at 18 months of age [12, 13]. Therefore, some studies have suggested that the selection based on SC must be performed at earlier ages to accelerate the genetic gain and to reduce the maintenance costs of non-productive animals [14]. Nevertheless, aspects related to the appropriate age for SC measurement for the selection of bulls still remain uncertain and need further investigation to improve the use of the SC in breeding programs of Zebu cattle [15].

Despite the positive correlation between SC and seminal parameters, this does not fully represent the reality of testicular parenchyma. Thus, an animal with suitable SC at an early age will not always show appropriate seminal quality at sexual maturity [16]. Nellore bulls of the same age and testicular size may provide different sperm motility; therefore, the semen production is also due to the testicular functionality and not only to the testis size [17].

Testicular growth description can be performed by fitting nonlinear regression models. These models enable to synthesize information from longitudinal size-age data in few parameters likely to be biologically interpretable [18]. Several nonlinear models such as Brody, von Bertalanffy, Logistic and Gompertz, have been used to describe SC growth curves [4, 19–21]. Nevertheless, other models such as Meloun, Michaelis-Menten and Hill remain overlooked. The Meloun model has been used to describe the growth of human fetuses [22], and due to its great flexibility this model is potentially useful for describing animal growth curves [23]. The Hill equation has been used to analyze quantitative drug-receptor relationship in pharmacology [24]; it also has been used for describing body growth curves in meat quails [25]. The Michaelis-Menten equation was originally used to relate the velocity of reaction to the amount of substrate [26]; its use in animal growth curves was already reported in beef cattle [27], dairy cattle [28] and pigs [29].

In general, random effects of experimental units must be modeled when using repeated measurements derived from the same animal over time. Thus, nonlinear mixed models considering both fixed and random effects simultaneously are preferred to better describe the observed data with individual repeated measures [30, 31].

In this context, we aimed to fit several nonlinear mixed models for SC measurements of Nellore bulls to choose one that would better predict testicular growth. Additionally, we compared the SC growth curve from divergently classified bulls under the initial hypothesis that young bulls (up to 21 months old) may reach similar values of SC at adult age (around 3–4 years old).

## Materials and methods

### Bulls and dataset

The data used in this study were from Nellore bulls raised in extensive management, born between 1997 and 2009. Animals were located in two farms. The first one is located in Magda/SP (20.6455° South, 50.2314° West), with an annual average temperature of 22.0°C and annual rainfall of 1200 mm; and the second one is located in Dois Irmãos do Buriti/MS (20.2947° South, 55.4454° West), with an annual average temperature of 23.3°C and annual rainfall of 1400 mm.

In both farms, the calving season occurs from August to November. After birth, calves are kept with their mothers in *Urochloa spp* pasture, with *ad libitum* water and mineral salt; weaning is performed when the calves reach 7–8 months old. Scrotal circumference (SC) was measured in the region of the largest diameter of the testes and included both testes positioned symmetrically side by side, leaving the skin of the scrotum distended. The SC measurements of the bulls were obtained annually at the time of the breeding soundness exam, which begins around 18 months of age. The ages at the moment of the SC measurement varied from 497 to 4340 days.

A total of 3,918 SC measurements from 843 bulls comprised the dataset. Table 1 shows the distribution of SC measurements according locate and age.

**Table 1:** Distribution of the numbers of scrotal circumference (SC) measurements by farm and age.

Farm	SC measurements (n)	Bulls (n)
SP	3265	701
MS	653	142
Age	SC measurements (n)	Bulls (n)
≤ 24 months	1017	843
> 24 - ≤ 48 months	777	510
> 48 months	2124	843

### Nonlinear models

Initially, estimates of SC growth curves were obtained by using nine nonlinear models (Table 2). Eight models are asymptotic, describing a growth that never exceeds a horizontal asymptote, whereas the Tanaka model allows an indeterminate growth without an asymptote [4, 20].

**Table 2:** Nonlinear models evaluated in this study to describe scrotal circumference (SC) growth in Nellore bulls.

Model	Equation
Brody [32]	${SC}_{t}=\beta_{1}(1-\beta_{2}e^{\left(-\beta_{3}t\right)})+e_{i}$
Gompertz [33]	${SC}_{t}=\beta_{1}e^{\left(-\beta_{2}e^{\left(-\beta_{3}t\right)}\right)}+e_{i}$
Hill [34]	${SC}_{t}=\frac{\beta_{1}t^{\beta_{3}}}{{\beta_{2}}^{\beta_{3}}+t^{\beta_{3}}}+e_{i}$
Logistic I [4]	${SC}_{t}=\frac{\beta_{1}}{1+\beta_{2}e^{\left(-\beta_{3}t\right)}}+e_{i}$
Logistic II [35]	${SC}_{t}=\frac{\beta_{1}}{1+e^{\left(\beta_{2}-\beta_{3}t\right)}}+e_{i}$
Meloun [22]	${SC}_{t}=\beta_{1}-\beta_{2}e^{\left(-\beta_{3}t\right)}+e_{i}$
Michaelis-Menten [36]	${SC}_{t}=\frac{\beta_{1}t}{t+\beta_{2}}+e_{i}$
Tanaka [4]	${SC}_{t}=(1/\sqrt{\beta_{2}})\ln\left(\left|2\beta_{2}\left(t-\beta_{4}\right)+2\sqrt{{\beta_{2}}^{2}{\left(t-\beta_{4}\right)}^{2}+\beta_{1}\beta_{2}}\right|\right)+\beta_{3}$
Von Bertalanffy [37]	${SC}_{t}=\beta_{1}{\left(1-\beta_{2}e^{\left(-\beta_{3}t\right)}\right)}^{3}+e_{i}$

*SC*_*t*_ = scrotal circumference at *t* days of age; *β*_1_ = asymptote (SC at maturity); *β*_2_ = integration constant, for the Michaelis Menten model *β*_2_ is the age where *SC* = *β*_1_/2; *β*_3_ = maturity index; *β*_4_ = inflection point.

In nonlinear models that describe SC growth curves, *SC*_*t*_ is the scrotal circumference at *t* days of age; *β*_1_ (asymptote) is the estimated SC at maturity; *β*_2_ is an integration constant important to shape the sigmoid curve but without biological interpretation, except for the Michaelis Menten model in which *β*_2_ is the age where *SC* = *β*_1_/2; *β*_3_ is a maturing index, establishing the earliness with which SC approaches the asymptote; and *β*_4_ is the inflection point in the Tanaka model. The inflection point is the time at which growth acceleration ends and the self-inhibition phase begins, until reaching SC size at maturity [4, 38, 39]. It is noteworthy that in the Tanaka model, *β*_4_ is the abscissa of the inflection point and it is the only parameter with biological interpretation in this model [4].

The inflection points and age at inflection point for the Gompertz, Hill, Logistic I and II, and von Bertalanffy models were calculated as showed in Table 3.

**Table 3:** Inflection points and age at inflection points.

Model	Inflection point (IP)	Age at IP
Gompertz [39]	${0.368\hat{\beta }}_{1}$	$\frac{\ln\left(\frac{\ln(0.368)}{{-\hat{\beta }}_{2}}\right)}{{-\hat{\beta }}_{3}}$
Hill [24]	$\frac{{\hat{\beta }}_{1}(\frac{{\hat{\beta }}_{3}-1}{{{\hat{\beta }}_{2}}^{{-\hat{\beta }}_{3}}\left({\hat{\beta }}_{3}+1\right)})}{{{\hat{\beta }}_{2}}^{{\hat{\beta }}_{3}}+(\frac{{\hat{\beta }}_{3}-1}{{{\hat{\beta }}_{2}}^{{-\hat{\beta }}_{3}}\left({\hat{\beta }}_{3}+1\right)})}$	${\left(\frac{{\hat{\beta }}_{3}-1}{{{\hat{\beta }}_{2}}^{{-\hat{\beta }}_{3}}\left({\hat{\beta }}_{3}+1\right)}\right)}^{\frac{1}{{\hat{\beta }}_{3}}}$
Logistic I [38]	$\frac{{\hat{\beta }}_{1}}{2}$	$\frac{\ln\left({\hat{\beta }}_{2}\right)}{{\hat{\beta }}_{3}}$
Logistic II [35]	$\frac{{\hat{\beta }}_{1}}{2}$	$\frac{-\beta_{2}}{-{\hat{\beta }}_{3}}$
von Bertalanffy [39]	${0.2963\hat{\beta }}_{1}$	$\frac{\ln\left(\frac{0.2963^{\frac{1}{3}}-1}{{-\hat{\beta }}_{2}}\right)}{{-\hat{\beta }}_{3}}$

${\hat{\beta }}_{1},{\hat{\beta }}_{2}$ and ${\hat{\beta }}_{3}$ are the parameters estimated by the nonlinear models. ${\hat{\beta }}_{1}$ = asymptote (SC at maturity); ${\hat{\beta }}_{2}$ = integration constant; ${\hat{\beta }}_{3}$ = maturity index.

The SC growth curve parameters (*β*_1_, *β*_2_, *β*_3_ and *β*_4_) were estimated by using the NLMIXED procedure of the Statistical Analysis System (SAS) [40]. The assumed individual random effects allowed each bull to have their own subject-specific asymptote and shape centered at *β*_1_ and *β*_2_. The residual errors were assumed to be independent and identically distributed according to normal distribution with mean zero and variance *σ*^2^ [41]. The convergence criteria were the defaults of the SAS for the dual Quasi-Newton algorithm [40]. Genetic relationship between individuals was not considered in the analysis.

### Goodness of fit

Goodness of fit of all models was evaluated according to the following criteria.

Akaike’s information criterion (AIC), given by *AIC* = −2*loglike* + 2*p*, where *loglike* is the logarithm of the maximum likelihood considering parameter estimates and *p* is the number of independently fitted parameters within the model. Lower values of AIC reflect a better fitting of the model [42]. Bayesian information criterion (BIC), given by *BIC* = −2*loglike* + *p* ln *n*, where *n* is the number of observations used to fit the curve. Lower values of BIC also reflect a better fitting of the model [43]. The adjusted R^2^, which determines the percentage of variation in SC measures explained by the statistical model, was calculated as: $R_{adj}^{2}=R^{2}-(\frac{p-1}{n-p})\times (1-R^{2})$, where $R_{adj}^{2}$ is the adjusted coefficient of determination; *R*^2^ is the square of the correlation coefficient between observed and predicted values; *p* is the number of parameters of the model; and *n* is the number of observations. Error sum of squares (ESS) was calculated as $ESS=\sum {\left(Y_{i}-{\hat{Y}}_{i}\right)}^{2}/n$, where the deviation of an observation *Y*_*i*_ was calculated from its own estimated mean ${\hat{Y}}_{i}$. The ESS is considered as an accepted control of fitting quality [4].

Percentage of convergence is used when individual fitting are obtained. Thus, it is possible to observe which model presents greater facility of convergence, which is given by the percentage of fitted models that converged. This percentage refers to animals whose convergence has been observed in up to 1000 iterations [44]. Moreover, animals that converged to unreal values were not considered as successful convergence.

### Comparisons among classifications

After the choice of the best model, bulls were divided into five groups according to the size of the SC in their first breeding soundness examination, which occurred until bulls were 21 months old, as showed in Table 4. Growth curves were estimated for each group.

**Table 4:** Distribution of the number of bulls and SC measurements according to classification in the first breeding soundness examination.

Classification^a^	Bulls (n)	SC measurements (n)
SC < 28 cm	24	119
28 cm ≤ SC < 30 cm	54	251
30 cm ≤ SC < 32 cm	183	857
32 cm ≤ SC < 34 cm	265	1225
SC ≥ 34 cm	317	1466

^a^Classification at 18–21 months old.

From the estimation of the individual curves, animals that reached convergence were used to create a new dataset with estimated parameters. Then, the effect of the classification of the SC size, farm (SP and MS) and year of birth (grouped in 1997–2003 and 2004–2009) on the parameters were evaluated by using the GLM procedure of the SAS, according to the model: *Y*_*ijkl*_ = *μ* + *C*_*i*_ + *F*_*j*_ + *B*_*k*_ + (*CF*)_*ij*_ + (*CB*)_*ik*_ + (*FB*)_*jk*_ + *e*_*ijkl*_, where *Y*_*ijkl*_ = response; *μ* = constant; *C*_*i*_ = effect of classification; *F*_*j*_ = effect of farm; *B*_*k*_ = effect of the year of birth; (*CF*)_*ij*_ = interaction between classification and farm; (*CB*)_*ik*_ = interaction between classification and year; (*FB*)_*jk*_ = interaction between farm and year; and *e*_*ijkl*_ = error. When normality of errors and homogeneity of variances were not achieved, data were submitted to the square root transformation. The least square means (LS-means) were compared by Tukey-Kramer test, assuming the level of significance equal to 0.05.

## Results

**Fig 1:**
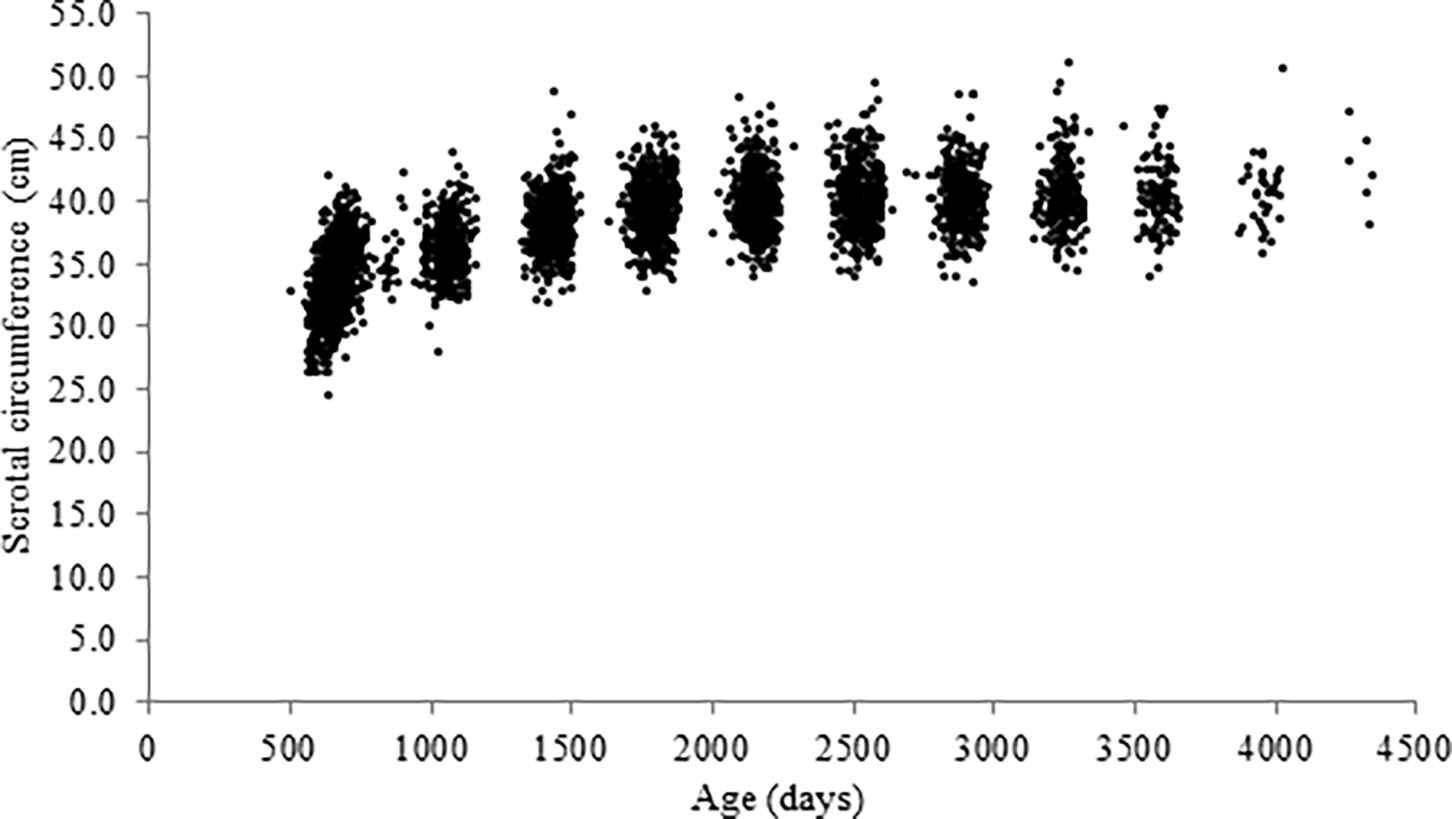
Observed scrotal circumference of Nellore bulls from 497 to 4340 days of age.

Observed data are showed in Fig 1. The SC measurements ranged from 24.5 cm to 51 cm over all analyzed periods.

Regarding the parameter estimates, the Hill and Michaelis-Menten models showed the highest asymptotic values, whereas both Logistic I and II models showed the lowest ones (Table 5).

**Table 5:** Parameter estimates (±SE) for nonlinear models used to describe scrotal circumference growth in Nellore bulls.

Model	${\hat{\beta }}_{1}$	${\hat{\beta }}_{2}$	${\hat{\beta }}_{3}$	${\hat{\beta }}_{4}$
Brody	41.1772 ± 0.1130	0.4141 ± 0.007429	0.001216 ± 0.000032	
Gompertz	41.0997 ± 0.1105	0.4853 ± 0.009292	0.001318 ± 0.000033	
Hill	43.5115 ± 0.2938	193.64 ± 5.2930	0.9882 ± 0.04006	
Logistic I	41.0284 ± 0.1082	0.5709 ± 0.01166	0.001422 ± 0.000034	
Logistic II	41.0285 ± 0.1082	-0.5606 ± 0.02041	0.001422 ± 0.000034	
Michaelis-Menten	43.4332 ± 0.1170	194.99 ± 2.6282		
Meloun	41.1774 ± 0.1130	17.0487 ± 0.2966	0.001216 ± 0.000032	
Tanaka	1099.83 ± 499.52	0.1466 ± 0.009771	21.7716 ± 0.4328	517.63 ± 16.1496
Von Bertalanffy	41.1240 ± 0.1112	0.1533 ± 0.002873	0.001284 ± 0.000033	

${\hat{\beta }}_{1}$ = asymptote, SC at maturity, except for Tanaka model; ${\hat{\beta }}_{2}$ = integration constant, except for Michaelis Menten model where ${\hat{\beta }}_{2}$ is the age where *SC* = ${\hat{\beta }}_{1}/2;\;{\hat{\beta }}_{3}$ = maturity index; ${\hat{\beta }}_{4}$ = inflection point.

None of the asymptotic models presented a reliable inflection point estimate. The Gompertz, Logistic I and II, and von Bertalanffy models presented inflection points equal to 15.1 cm, 20.5 cm, 20.5 cm, and 12.2 cm, respectively. However, all these models estimated negative values for age at inflection point. The Hill model presented a ${\hat{\beta }}_{3}$ value equal to 0.9882, but this model has an inflection point only when *β*_*3*_ is higher than 1 [24].

The Tanaka model estimated the inflection point for SC at 517.63 days with 30.2 cm. Even though these values are reliable, it is noteworthy that *β*_*4*_ is the only parameter with biological interpretation in the Tanaka model, which limits its application to describe SC growth curves.

**Fig 2:**
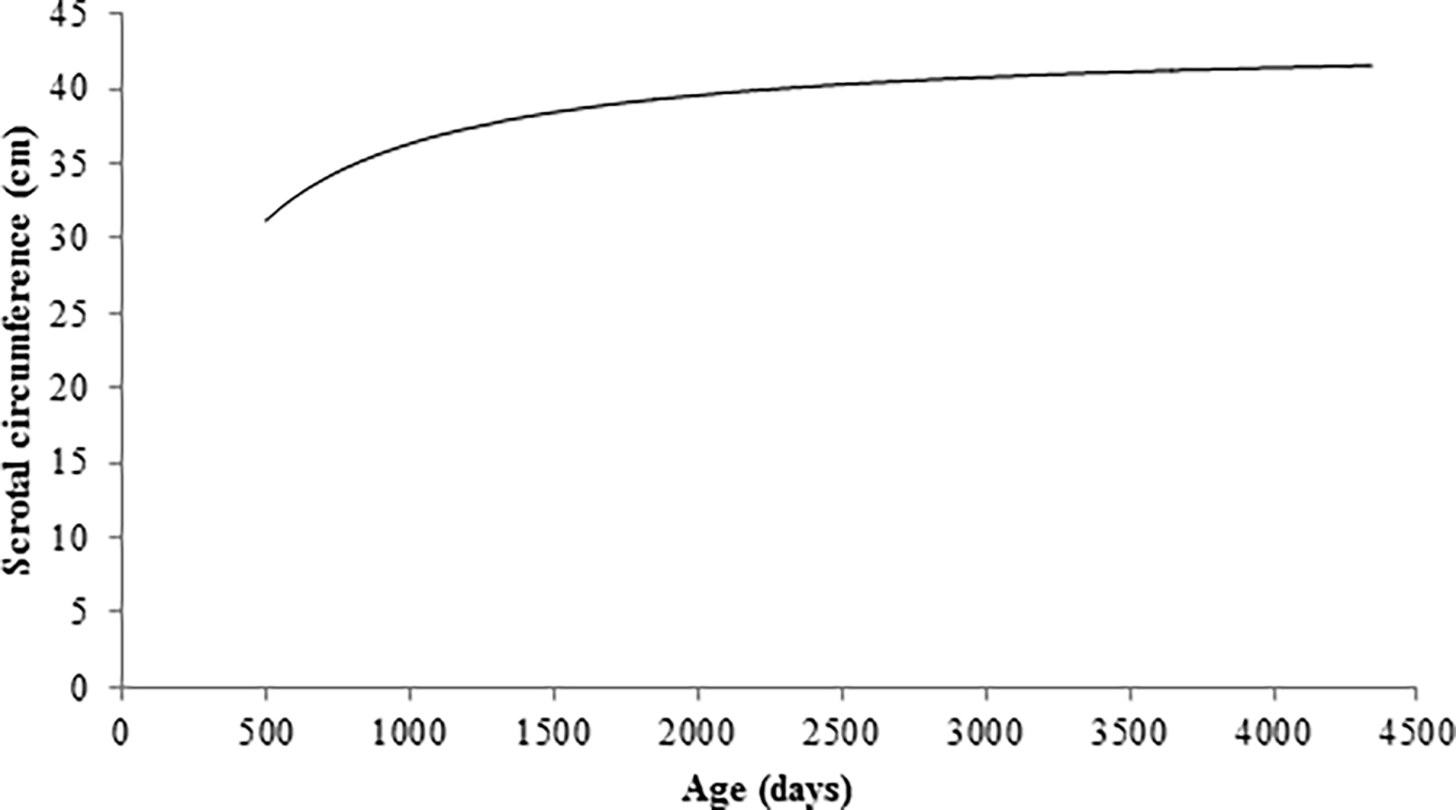
Scrotal circumference growth curve estimated by Michaelis-Menten model in Nellore bulls.

Goodness of fit for all models are shown in Table 6. Michaelis-Menten model showed the lowest values for Akaike’s information criterion (AIC) and Baeysian information criterion (BIC), indicating a better fitting. The adjusted R^2^ values were very similar among all models, with Tanaka being slightly higher followed by Michaelis-Menten model. For error sum of squares, Tanaka and Michaelis-Menten models showed the lowest values. Nevertheless, the Michaelis-Menten model presented a percentage of convergence higher than other models. This criterion is important for the study of individual growth curves because if a model presents high fitting quality but low percentage of convergence, just few animals could be used in the breeding program [44]. The SC growth curve estimated by Michaelis-Menten model is shown in Fig 2.

**Table 6:** Goodness of fit for nonlinear models used to describe scrotal circumference growth in Nellore bulls.

Model	AIC	BIC	Adj. R^2^	ESS	Conv. (%)
Brody	15,358	15,392	0.5804	5.9443	64.41
Gompertz	15,366	15,399	0.5804	5.9453	65.84
Hill	15,359	15,392	0.5805	5.9442	53.86
Logistic I	15,374	15,407	0.5803	5.9463	65.72
Logistic II	15,374	15,407	0.5803	5.9463	67.26
Meloun	15,358	15,392	0.5804	5.9443	61.21
Michaelis-Menten	15,357	15,385	0.5808	5.9417	96.32
Tanaka	15,705	15,743	0.5816	5.9269	53.74
Von Bertalanffy	15,363	15,396	0.5804	5.9450	65.72

AIC = Akaike’s information criterion; BIC = Bayesian information criterion; Adj. R^2^ = Adjusted coefficient of determination; ESS = Error sum of squares; Conv. = Percentage of convergence.

**Fig 3:**
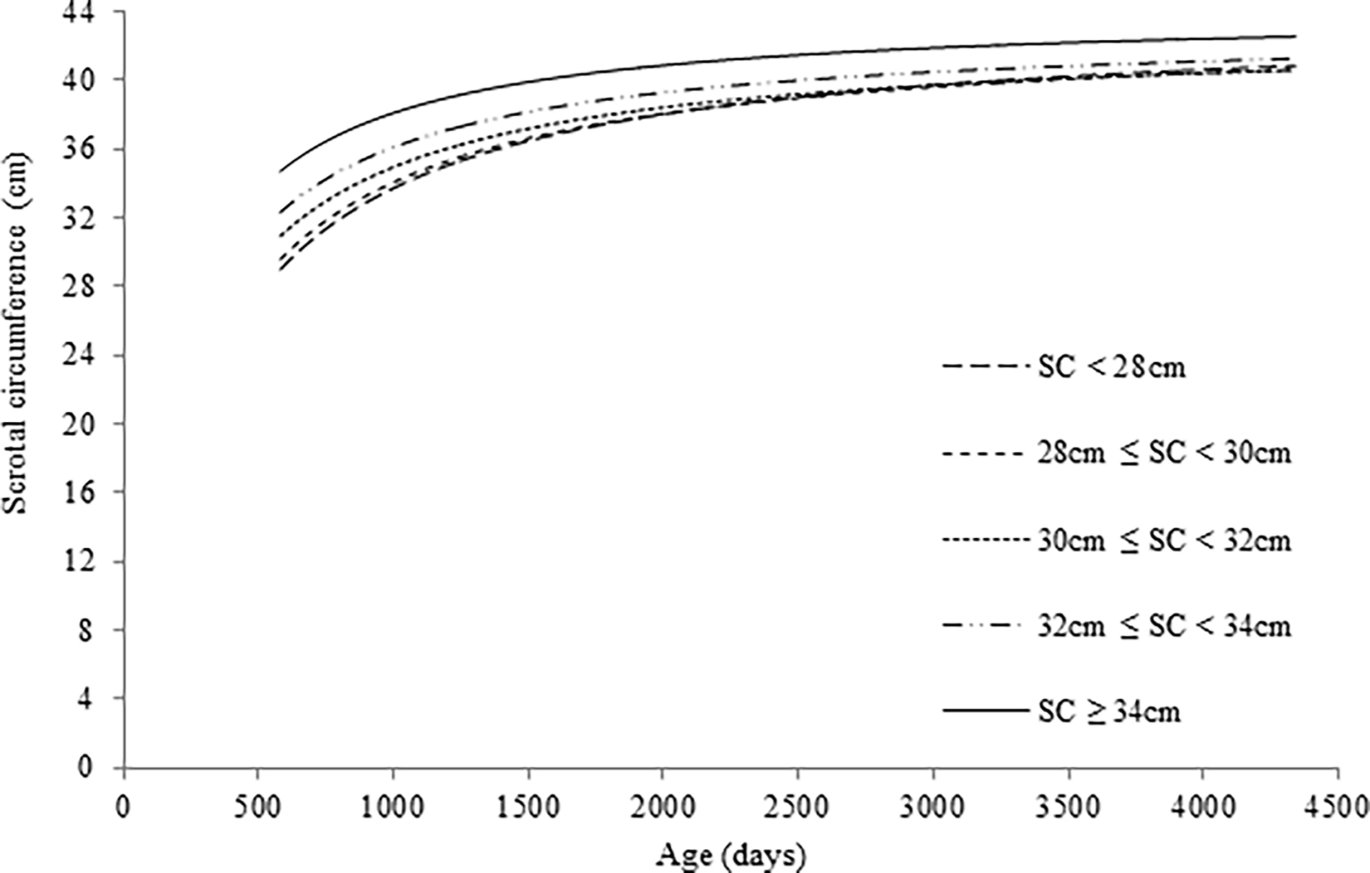
Scrotal circumference (SC) growth curves estimated by Michaelis-Menten model in Nellore bulls classified according SC size at first breeding soundness examination.

Based on goodness of fit measures, Michaelis-Menten model was selected for subsequent analyses. Parameters estimates for each group are shown in Table 7, and the growth curves estimated from this model are shown in Fig 3.

**Table 7:** Parameter estimates (±SE) by Michaelis-Menten model used to describe scrotal circumference growth in Nellore bulls classified according SC size at age of 18–21 months.

Classification	${\hat{\beta }}_{1}$	${\hat{\beta }}_{2}$
SC < 28 cm	43.5757 ± 0.8046	292.38 ± 18.1522
28 cm ≤ SC < 30 cm	43.0875 ± 0.4636	265.31 ± 9.8585
30 cm ≤ SC < 32 cm	42.6220 ± 0.2512	219.75 ± 5.2765
32 cm ≤ SC < 34 cm	43.0775 ± 0.2135	193.34 ± 4.3709
SC ≥ 34 cm	44.0659 ± 0.1765	156.86 ± 3.2838

${\hat{\beta }}_{1}$ = asymptote (SC at maturity); ${\hat{\beta }}_{2}$ = integration constant, it represents the age (in days) where $SC=\frac{{\hat{\beta }}_{1}}{2}$.

In Fig 3, it is possible to note that bulls with SC larger than 34 cm presented larger predicted testis size over all analyzed periods. On the other hand, bulls of the smallest SC have showed predicted SC similar to intermediate groups (28 cm ≤ SC < 32 cm), around 1200 days of age. This finding suggests that bulls that were classified as improper for reproduction at age of 18–21 months, in their adult life, can reach a similar condition to those bulls considered as good condition.

For Michaelis-Menten model, *β*_2_ is the age where *SC* = *β*_1_/2; thus, this parameter may be indicative of earliness of the testis development. In this sense, bulls with larger SC at 18–21 months of age presented lower values of *β*_2_ indicating precocity in the testis growth (Table 7).

From the estimation of the individual curves, animals that reached convergence were used to create a new dataset with individual estimated parameters to analyze the effects of the classification, farm, and year of birth on parameters ${\hat{\beta }}_{1}$ and ${\hat{\beta }}_{2}$. The effect of year of birth was not significant for any parameter (P > 0.05). There was no effect of farm and classification on asymptotic SC (P > 0.05). Nevertheless, for ${\hat{\beta }}_{2}$, classification (Table 8) and interaction between classification and farm (Table 9) showed significant effects (P < 0.05), but there was no difference between farms (P > 0.05).

**Table 8:** LS-means (±SE) of the parameters estimated by Michaelis-Menten model for each classification.

Classification	${\hat{\beta }}_{1}$	${\hat{\beta }}_{2}$
SC < 28 cm	43.28 ± 0.67 ^a^	302.48 ± 13.38 ^a^
28 cm ≤ SC < 30 cm	42.59 ± 0.46 ^a^	254.51 ± 9.72 ^ab^
30 cm ≤ SC < 32 cm	42.51 ± 0.23 ^a^	223.52 ± 4.76 ^b^
32 cm ≤ SC < 34 cm	43.19 ± 0.19 ^a^	203.56 ± 3.82 ^b^
SC ≥ 34 cm	43.37 ± 0.16 ^a^	146.05 ± 3.08 ^c^

Different letters in the same column indicate significant difference at P < 0.05. ${\hat{\beta }}_{1}$ = asymptote (SC at maturity); ${\hat{\beta }}_{2}$ = integration constant, it represents the age (in days) where $SC=\frac{{\hat{\beta }}_{1}}{2}$.

**Table 9:** Interaction between farm and classification for ${\hat{\beta }}_{2}$ (mean ± SE).

Classification	Farm	
	SP	MS
SC < 28 cm	318.02 ± 14.95 ^aA^	287.33 ± 31.79 ^aA^
28 cm ≤ SC < 30 cm	269.69 ± 10.36 ^aA^	239.78 ± 24.13 ^aA^
30 cm ≤ SC < 32 cm	221.02 ± 5.34 ^bA^	226.03 ± 10.46 ^aA^
32 cm ≤ SC < 34 cm	192.48 ± 4.08 ^cA^	214.95 ± 10.09 ^aA^
SC ≥ 34 cm	156.82 ± 3.44 ^dA^	135.67 ± 6.76 ^bA^

Different lowercase letters in the same column and uppercase letters in the same row indicate significant difference at P < 0.05.

## Discussion

Several authors have reported the use of nonlinear models to describe the growth curve of scrotal circumference on domestic animals. These authors have focused in some nonlinear models such as Brody, von Bertalanffy, Logistic and Gompertz [4, 19–21]; meanwhile other models remained overlooked. In this study, the Michaelis-Menten equation showed the best fitting among asymptotic models, especially regarding the percentage of convergence, an important criterion for studies of individual growth curves. The percentage of convergence indicates the number of animals to be used in an animal breeding program [44].

In several nonlinear models used to describe the SC growth curve, the *β*_*2*_ parameter is an integration constant without biological interpretation; however, in the Michaelis-Menten model, this parameter represents the age in days of half final growth [26]. Thus, in this study *β*_*2*_ may be considered as indicative of sexual precocity based on SC.

In breeding programs of Nellore breed, the measurement of the SC is usually performed up to age of 24 months, starting mostly at 18 months [12, 45]. The idea of performing the selection based on SC in earlier ages is to accelerate genetic gain [14]; however, Barth and Ominski [46] pointed out that SC measurement in weaned bulls (8 months of age) may not be a useful culling tool for breeding programs, due a large portion of bulls that did not met the minimum requirements for SC size at 240 days achieved these requirements at 365 days of age. The similarity of estimated SC among bulls of the lowest SC group and bulls with SC ranging from 28 to 32 cm (intermediate groups) around 1200 days of age is an important finding, since Zebu bulls, in tropical environments, start to be used for natural service in breeding seasons at 36–40 months of age. Nevertheless, it is noteworthy that the bulls with SC larger than 34 cm at 18–21 months old presented the highest values of SC along the most evaluated period, indicating a high potential, since SC is related to reproductive performance of males and females [6–8].

Nevertheless, in Nellore cattle, SC presented negative genetic correlation (-0.16) with birth weight, and positive but low magnitude genetic correlations with weight gain from weaning to yearling (0.12) [47] and post-weaning weight gain (0.189) [8]. Besides, genetic and phenotypic correlations near to zero were reported for rump fat and SC measured at 365 and 450 days, indicating that selection for SC at earlier ages would promote slow improvement in carcass traits [48, 49], and Marques et al. [50] reported low and negative genetic correlations between SC and daily weight gain (-0.11), and subcutaneous fat thickness (-0.04). Therefore, the similarity of the asymptotic values among all groups indicates that a selection at earlier ages may exclude animals with suitable genetic potential from the breeding program, including animals with suitable estimated breeding values (EBV) for other traits such as birth weight, post-weaning weight gain and carcass traits.

According to Fortes et al [45] larger SC at a young age contributes to lower rates of sperm DNA damage in Brahman bulls. Negative correlation between sperm DNA fragmentation and SC measured at 12, 18 and 24 but not at 6 months of age were reported by the authors, despite correlations were weak (ranging from -0.18 to -0.25). Nevertheless, Nellore young bulls (up to 24 months old) demonstrated to have spermatozoa in suboptimal condition in terms of nuclear integrity when compared to bulls with 3.5–7 years of age, suggesting a state of nuclear fragility or immaturity [51]. Moreover, some parsimony about SC values should be taken since the SC does not exactly represent the reality of testicular parenchyma. In this context, a bull with large SC at an early age will not always show suitable seminal parameters at sexual maturity [16]. Furthermore, semen production is also due to the testicular functionality and not only to the testis size [17].

The similarity observed in asymptotic values among all groups of this study was not found for *β*_*2*_ values. This finding suggests that the major difference among groups is related to the earliness in which the animal reaches a suitable value of SC. Several factors can influence sexual precocity in the Nellore bulls such as nutritional and genetic aspects [12].

According to literature reports, the heritability for SC in different ages in Nellore breed indicates a high genetic variability from 12 to 21 months (heritability ranging from 0.43 to 0.49), suggesting that the SC in young animals (12–15 months old) may be used as selection criterion allowing the early discard of animals that might not show suitable sperm production [14, 52]. Nevertheless, the correlation between SC in different ages and sexual precocity in females is fundamental to define the most appropriate age to measure this trait [12, 14, 53].

In Nellore cattle, genetic correlations between SC and reproductive traits of females have been reported with conflicting results. Negative genetic correlation (-0.40) between SC at 13–18 months (corrected to 15 months) and age at first calving (AFC) of females mated or inseminated at 14 months old [54], and positive genetic correlation (0.20) between SC and probability of pregnancy at 14 months (PP14) [55] have been described. However, genetic correlations close to zero were also reported between SC at 18 months and AFC and PP14 [56]. Grossi et al. [57] reported genetic correlation estimates of low magnitude for AFC and SC measured at 365 (0.10), 450 (-0.13), 550 (-0.13) and 730 (0.06) days of age, thus indicating that male selection for SC will not cause genetic changes to the AFC. On the other hand, Santana Jr et al. [58] reported that genetic correlation between AFC of heifers exposed to a bull at 24 months old and SC measured at several ages (from 400 to 654 days) presented lower variation (from -0.20 to -0.30), but the genetic correlation between SC and AFC of heifers exposed to a bull at 14 months was stronger (-0.70) and more negative at an early age of the SC measurement.

Additionally, moderate genetic correlations between SC at 18 months and heifer pregnancy (0.29) and stayability for five years (0.19) were reported suggesting that SC at 18 months could be incorporated in multitrait analysis to improve the prediction accuracy for heifer pregnancy merit of young bulls [59].

Therefore, genetic correlations between SC and female reproductive traits may be influenced by the age of measurement of the SC [12, 54, 60]. In Nellore cattle, SC measured at 12 months showed favorable correlation with the first date of calving while SC measured at 18 months presented unfavorable correlation; however, for first calving interval, SC measured at 18 months was favorably correlated with higher magnitude than SC measured at 12 months [60]. Thus, further investigations involving the female reproductive traits such as AFC, PP14, heifer pregnancy, stayability and SC evaluated in different ages are needed to establish the most suitable period to estimate the genetic correlations.

Sperm production may depend on other factors than SC, such as testicular volume and shape [61]. Thus, these traits should be considered in selection of bulls [62, 63]. Once there is a predominance of long shape testes in Zebu bulls, animals with long testes can be discarded due to present smaller SC than their contemporary with oval testes [56, 64]. Moreover, there are evidences that testicular shape can change with age with reduction of the long shape frequency from 12 to 18 months of age [63].

Results obtained in the present study showed that young Nellore bulls with different testicular size reach similar SC values in adult life; the differences found in growth curves are related to sexual precocity of each individual. Since the most precise age for selection of young bulls based on SC value still remains unclear, it seems that for Nellore bulls, selection at too early ages, even at 18 months, may lead to discard bulls with suitable reproductive potential.

## Supporting information

S1 Fig Boxplot of the monthly rainfall of the farm SP (region 107) from 1981 to 2010 (black line) and 2011 (red line). Available from: http://clima1.cptec.inpe.br/evolucao/pt.
(DOCX)

S2 Fig Monthly temperatures (minimum and maximum) of the farm SP (Last 30 years). Available from: https://www.climatempo.com.br/climatologia/2381/magda-sp.
(DOCX)

S3 Fig Boxplot of the monthly rainfall of the farm MS (region 105) from 1981 to 2010 (black line) and 2011 (red line). Available from: http://clima1.cptec.inpe.br/evolucao/pt.
(DOCX)

S4 Fig Monthly temperatures (minimum and maximum) of the farm MS (Last 30 years). Available from: https://www.climatempo.com.br/climatologia/4987/doisirmaosdoburiti-ms.
(DOCX)

S5 Fig Scrotal circumference growth curves of Nellore bulls estimated by Brody, Gompertz, Logistic I and II and von Bertalanffy models. (DOCX)

S6 Fig Scrotal circumference growth curves of Nellore bulls estimated by Hill, Meloun, Michaelis-Menten and Tanaka models. (DOCX)
