# Supplementary material for: Can scrotal circumference-based selection discard bulls with good productive and reproductive potential?
Source: PLoS One. 2018 Mar 1;13(3):e0193103. doi: 10.1371/journal.pone.0193103 (PMC5832217; doi:10.1371/journal.pone.0193103)
Supplement: S1 Fig — Available from: http://clima1.cptec.inpe.br/evolucao/pt. (DOCX) [file pone.0193103.s001.docx]

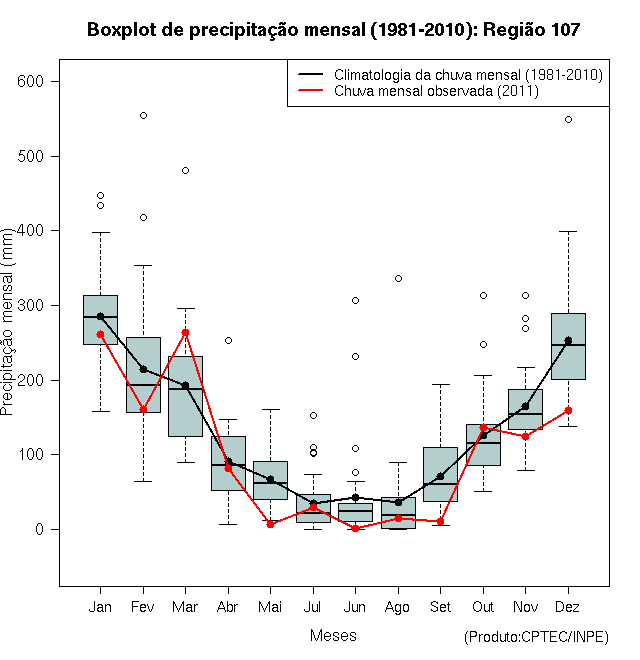


Supplementary figure 1. Boxplot of the monthly rainfall of the Farm SP (Region 107) from 1981 to 2010 (black line) and 2011 (red line). Available from: <http://clima1.cptec.inpe.br/evolucao/pt>
