# Supplementary material for: Can scrotal circumference-based selection discard bulls with good productive and reproductive potential?
Source: PLoS One. 2018 Mar 1;13(3):e0193103. doi: 10.1371/journal.pone.0193103 (PMC5832217; doi:10.1371/journal.pone.0193103)
Supplement: S4 Fig — Available from: https://www.climatempo.com.br/climatologia/4987/doisirmaosdoburiti-ms. (DOCX) [file pone.0193103.s004.docx]

Supplementary figure 4. Monthly temperatures (minimum and maximum) of the Farm MS (Last 30 years).

Available from: <https://www.climatempo.com.br/climatologia/4987/doisirmaosdoburiti-ms>
