# Supplementary material for: Can scrotal circumference-based selection discard bulls with good productive and reproductive potential?
Source: PLoS One. 2018 Mar 1;13(3):e0193103. doi: 10.1371/journal.pone.0193103 (PMC5832217; doi:10.1371/journal.pone.0193103)
Supplement: S6 Fig — (DOCX) [file pone.0193103.s006.docx]

Supplementary figure 6. Scrotal circumference growth curves of Nellore bulls estimated by Hill, Meloun, Michaelis-Menten and Tanaka models.
